# Exploring the Therapeutic Potential of Lenvatinib: A Complete Pathological Response in an Operable Case of Hepatocellular Carcinoma

**DOI:** 10.7759/cureus.64166

**Published:** 2024-07-09

**Authors:** Vipul Goyal, Madhu Muralee, Subhash Raveendran, Chandramohan K, Anoop T M

**Affiliations:** 1 Surgical Oncology, Regional Cancer Centre, Thiruvananthapuram, IND; 2 Medical Oncology, Regional Cancer Centre, Thiruvananthapuram, IND

**Keywords:** neoadjuvant treatment, pathological complete response (pcr), hepatocellular carcinoma, operable, lenvatinib

## Abstract

Hepatocellular carcinoma (HCC) is a leading cause of cancer-related mortality worldwide, with increasing incidence and mortality rates. This case report presents a unique instance of a 66-year-old male patient with operable HCC who achieved a complete pathological response after short-term preoperative treatment with lenvatinib. The patient, with a history of diabetes and hypertension, was diagnosed with HCC and started on lenvatinib due to logistical reasons. Despite discontinuing the treatment after one week due to altered sensorium, a significant reduction in tumor size was observed. The patient underwent successful surgery, and the final histopathology report indicated a complete pathological response. This case highlights the potential of lenvatinib as a therapeutic option in the management of HCC, even in operable cases, and opens avenues for further research into its efficacy and applicability.

## Introduction

Hepatocellular carcinoma (HCC) is a significant global health concern, being one of the primary causes of cancer-related mortality. The incidence and mortality rates are projected to increase by over 55% by 2040 [1]. The etiology of HCC is predominantly associated with cirrhosis, hepatitis B virus (HBV), and hepatitis C virus (HCV).

The prognosis of HCC, as per the 2022 update of the Barcelona Clinic for Liver Cancer (BCLC), is determined based on tumor burden, liver function, and physical status. These factors are further assessed by various indices and scores such as alfa fetoprotein (AFP), albumin-bilirubin index (ALBI) score, Child-Pugh score, and model for end-stage liver disease (MELD) [2].

Lenvatinib, a multi-targeted drug, acts on various targets, including VEGFR 1-3, PDGFR α, KIT, RET, and FGFR 1-4. Prior to 2018, sorafenib was the preferred choice for treating advanced/unresectable HCC. However, the REFLECT trial (A Multicenter, Randomized, Open-Label, Phase 3 Trial to Compare the EFficacy and Safety of LEnvatinib (E7080) Versus Sorafenib in First-Line Treatment of Subjects With UnreseCtable HepaTocellular Carcinoma), a prospective randomized phase III study in 2018 demonstrated the non-inferiority of lenvatinib to sorafenib, with a median overall survival of 13.6 months for lenvatinib versus 12.3 months for sorafenib (HR 0.92, 95% CI: 0.79-1.06). Consequently, lenvatinib was approved for the treatment of unresectable and advanced HCC [3].

Our tertiary care center, located in the south of India, serves the population of Kerala and the border areas of Tamil Nadu. Logistical considerations play a significant role in formulating and deciding a treatment plan for patients seeking treatment at our center, including the unavailability of dates for surgery due to the long waiting period. We report a unique case where lenvatinib was used for one week in an operable case of HCC, leading to a complete pathological response. This case underscores the potential of lenvatinib as a therapeutic option in the management of HCC.

## Case presentation

**Figure 1:**
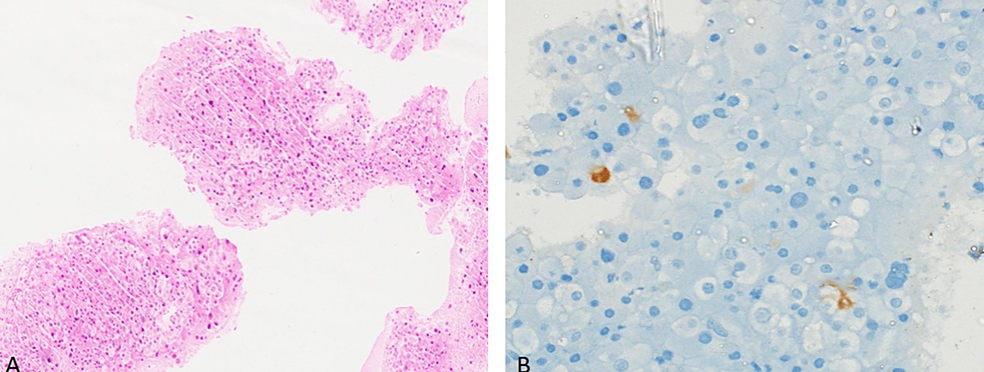
A: Fragments showing neoplasm composed of hepatoid cells with moderate nuclear pleomorphism, vesicular nuclei, and moderate eosinophilic cytoplasm; B: HepPar1-positive in occasional cells

The patient, a 66-year-old male with a history of diabetes mellitus and systemic hypertension, presented with abdominal pain. Upon evaluation, he was diagnosed with HCC, confirmed by biopsy (Figure 1).

**Figure 2:**
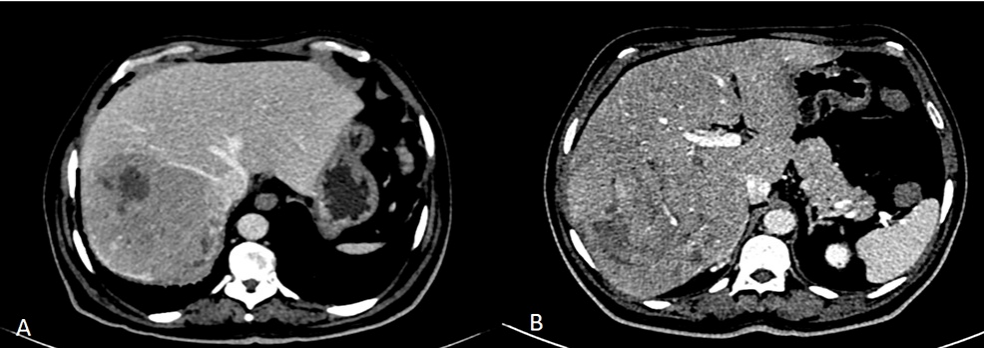
Preoperative CECT A: right hepatic vein abutting the tumor; B: right posterior branch of the right portal vein going into the tumor CECT: contrast-enhanced computed tomography

A triple-phase contrast-enhanced CT scan revealed a 14.6 x 11.4 x 9.3 cm lesion in segments VI and VII of the liver, with arterial enhancement and venous washout. The right posterior branch of the portal vein showed an abrupt cut-off without main branch thrombosis (Figure 2).

He had features of very early cirrhosis with no portal hypertension, tested negative for viral markers of hepatitis, the serum AFP value was 3 ng/ml, the Child-Pugh score was class A (score 5), and he had an ALBI grade of 1, indicating well-compensated cirrhosis and a low risk of liver-related mortality.

**Figure 3:**
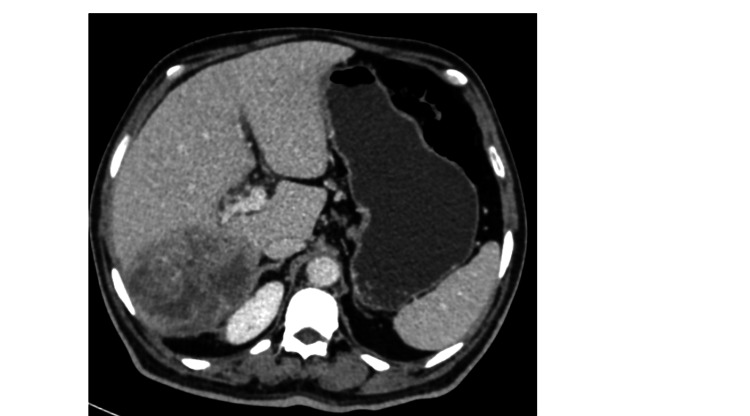
CECT images showing interval regression of the tumor CECT: contrast-enhanced computed tomography

Preoperatively, the patient was started on lenvatinib due to logistic reasons. The usual lenvatinib dose is 12 mg/day orally but our patient was started on 8 mg/day and was planned for dose escalation depending on dose toleration. However, after two weeks, he developed an altered sensorium and discontinued the treatment. A post-neoadjuvant chemotherapy re-evaluation scan showed an interval reduction in the size of the tumor to 10.4 x 9.6 x 7.4 cm, with reduced enhancement and increased necrotic area (Figure 3). The patient was planned for a right lateral sectionectomy, six weeks post the last dose of lenvatinib. According to the mRECIST (modified Response Evaluation Criteria in Solid Tumors) score, it was a stable disease.

**Figure 4:**
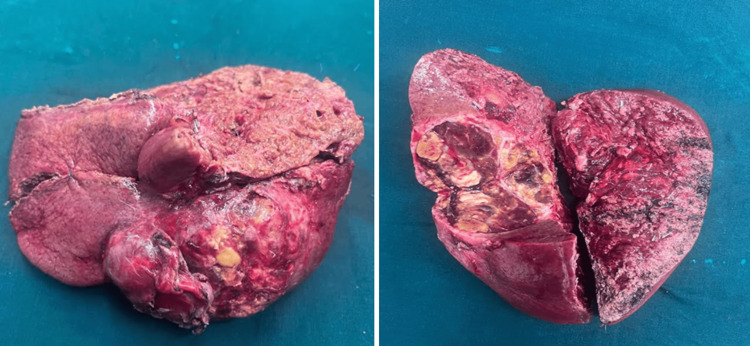
Resected specimen of right hepatectomy

Intraoperatively, the tumor was found to be extending into segment VIII with posterior adherence to the right adrenal gland and diaphragm. He underwent a formal right hepatectomy with en-bloc resection of the right adrenal gland and a part of the diaphragm (Figure 4).

**Figure 5:**
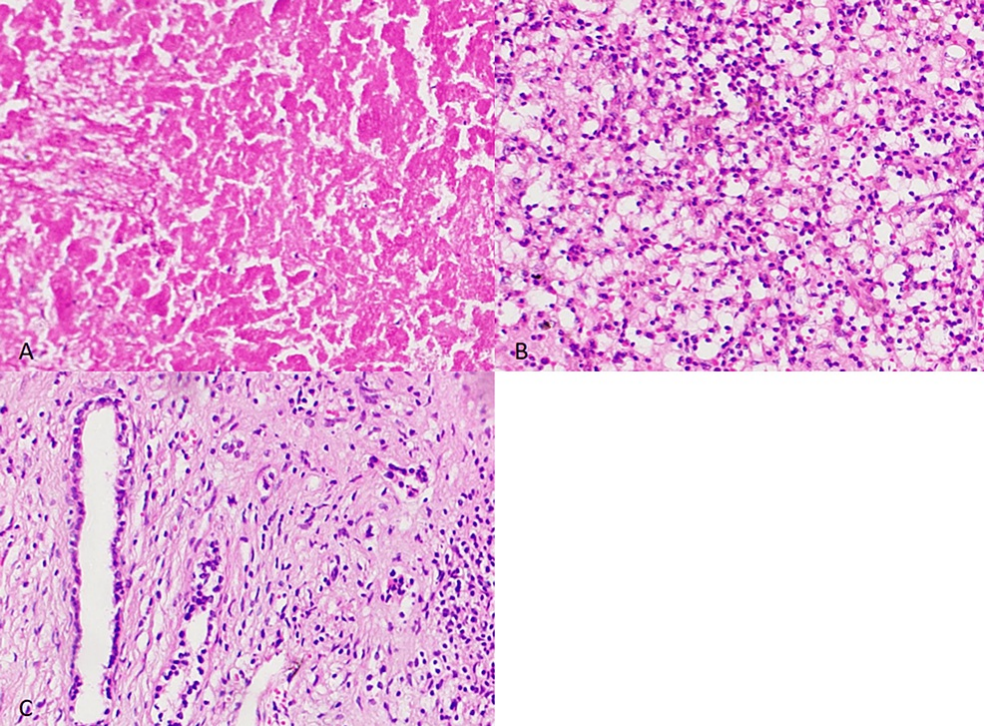
Post-right hepatectomy specimen showing no viable tumor cells seen with therapy-related changes A: areas of necrosis; B: collection of foamy cells and inflammatory cells; C: collection of foamy cells and inflammatory cells

The patient’s postoperative course was uneventful, and he was discharged a week after surgery. His final histopathology report indicated a complete pathological response, suggesting that all detectable cancer was eliminated by the treatment (Figure 5). This is a promising outcome, as it is associated with improved survival rates in patients with HCC. Currently, the patient is under follow-up with disease-free survival (DFS) of 10 months.

## Discussion

The mortality of HCC is related to the aggressive nature of the disease and the delayed presentation at a stage where radical treatment of resection is not possible and the other treatment option, transplantation, is limited by logistics. Thus, it is imperative to have an effective systemic treatment. Sorafenib was the first step in that direction followed by the recent use of lenvatinib and other drugs, including atezolizumab-bevacizumab (PDL-1 inhibitor-VEGF inhibitor) and durvalumab-tremelimumab (PDL-1 inhibitor-CTLA-4 inhibitor), which are currently the standard of care in patients with advanced HCC with preserved liver function status.

HCC is associated with cirrhotic liver, which may be due to alcohol consumption, non-alcoholic steatohepatitis, and HBV and HCV infection. Our patient has had a history of alcohol consumption, which he discontinued three years ago. He tested negative for viral markers assays.

The effect and tolerance of systemic treatment fares according to the performance status and liver function, which can be measured using various scores such as the ALBI score and the Child-Turcotte-Pugh (CTP) score. Our patient had an ALBI grade of 1, MELD score of 6, CTP score of A, and Eastern Cooperative Oncology Group (ECOG)-performance status (PS) of 1, which translates into better tolerability to the treatment. Our patient fulfilled the inclusion criteria of the REFLECT trial except that he belonged to BCLC stage A. 

Lenvatinib is associated with multiple side effects and hypertension is the most common side effect followed by diarrhea, decreased appetite, and decreased weight. It is also known to cause hepatic failure in patients. In the REFLECT trial, only 15% of patients could complete the planned therapy and most of the patients developed treatment-associated side effects in 1-2 months of therapy initiation (3). Our patient was already diagnosed with hypertension and in one week of therapy, he developed an altered sensorium for which the treatment was discontinued.

In the literature, sparse data are available regarding the pathological complete response after lenvatinib therapy. In one case series, two patients had shown a pathological complete response (pCR) after lenvatinib, which was administered for six months, but both patients had received conventional-transcatheter arterial embolization (cTAE) [4]. One patient had shown pCR after receiving lenvatinib for one year in a patient having unresectable HCC [5].

Just after two weeks of therapy, our patient had developed a pCR with few case reports from around the world; it raises the question of the role of lenvatinib in an operable HCC where downsizing may preserve the future liver remnant, preserving the nearby vessels, and the field sterilization leading to margin-negative resections.

## Conclusions

The presented case offers insights into the dynamic interplay between systemic therapy and surgical intervention in the management of HCC. It underscores the need for tailored therapeutic approaches and collaborative decision-making to optimize outcomes in this challenging oncological scenario. Further studies are warranted to elucidate the precise role of lenvatinib in enhancing the feasibility and efficacy of surgical resection in HCC.
